# Validation of internal reference genes for quantitative real-time PCR in a non-model organism, the yellow-necked mouse, ***Apodemus flavicollis***

**DOI:** 10.1186/1756-0500-2-264

**Published:** 2009-12-23

**Authors:** Jan Axtner, Simone Sommer

**Affiliations:** 1 Leibniz Institute for Zoo and Wildlife Research (IZW), Evolutionary Genetics, Alfred-Kowalke-Strasse 17 10315 Berlin, Germany

## Abstract

**Background:**

Reference genes are used as internal standards to normalize mRNA abundance in quantitative real-time PCR and thereby allow a direct comparison between samples. So far most of these expression studies used human or classical laboratory model species whereas studies on non-model organism under in-situ conditions are quite rare. However, only studies in free-ranging populations can reveal the effects of natural selection on the expression levels of functional important genes. In order to test the feasibility of gene expression studies in wildlife samples we transferred and validated potential reference genes that were developed for lab mice (*Mus musculus*) to samples of wild yellow-necked mice, *Apodemus flavicollis*. The stability and suitability of eight potential reference genes was accessed by the programs BestKeeper, NormFinder and geNorm.

**Findings:**

Although the three programs used different algorithms the ranking order of reference genes was significantly concordant and geNorm differed in only one, NormFinder in two positions compared to BestKeeper. The genes ordered by their mean rank from the most to the least stable gene were: *Rps18*, *Sdha*, *Canx*, *Actg1*, *Pgk1*, *Ubc*, *Rpl13a *and *Actb*. Analyses of the normalization factor revealed best results when the five most stable genes were included for normalization.

**Discussion:**

We established a SYBR green qPCR assay for liver samples of wild *A. flavicollis *and conclude that five genes should be used for appropriate normalization. Our study provides the basis to investigate differential expression of genes under selection under natural selection conditions in liver samples of *A. flavicollis*. This approach might also be applicable to other non-model organisms.

## Background

Quantitative real-time RT PCR (qPCR) has become a tool with a broad spectrum of use in molecular biology [1]. By quantifying mRNA levels it allows valuable insights into the variation of gene expression between certain individuals or different treatment groups. The most common practice in qPCR is the relative measurement of the expression of a gene of interest after normalization to an internal reference gene. These formerly called house-keeping genes were thought to be constantly expressed in every cell or every tissue and were supposed to be neither up nor down regulated. This assumption has proven false by a growing number of studies [2-4]. All genes seem to be regulated under some conditions and there seems to be no universal reference gene with a constant expression in all tissues [5-9]. But still the relative quantification against an internal reference gene is the most accurate way to detect expression differences especially in low copy mRNA because it controls for artificial variation, e.g. due to differences in the amount of sample, RNA extraction or reverse transcription efficiency [10]. Thus, a careful validation of the usefulness of potential reference genes is highly recommended [1,6,10-15] but not always applied [16]. So far gene expression studies and therefore also reference gene validations are mainly limited to human or classical laboratory organisms as non-model species often suffer from the lack of background information available. For example the real-time PCR primer data base RTPrimerDB [17] includes 5319 primer sets for animals and humans, whereof 3992 were designed for humans followed by 805 for mice (*Mus musculus*) and 454 for rats (*Rattus norvegicus*) commonly used in labs. But particularly non-model species are of great interest to evolutionary genetics or ecologists as classical model species might be poor reflections of wildlife which face the constantly changing and challenging conditions of their natural environment [18]. Focusing just on model species could mean working on the expense of ecological and evolutionary realism and in-situ studies on wild populations are required to account for natural selection conditions.

In this study we established a SYBR green qPCR assay for liver samples obtained from wild caught *Apodemus flavicollis*. The yellow-necked mouse is a common European murid in deciduous and mixed forests. It belongs to the subfamily Murinae [19] and has been subject to a broad range of genetic, ecological, evolutionary and parasitological studies [20-25]. Especially host-parasite interactions are of special interest in this species as this species serve as one of the main reservoir for vector-borne diseases agents (e.g. *Salmonella *spp., Borreliosis or Hanta virus infections) in Central Europe [25]. The results of our study are the prerequisite to investigate the adaptive variance of expression levels of immune genes, specifically major histocompatibility complex class II genes, in relation to individual pathogen burden to test the hypothesis that in a natural environment not only structural sequence variation but also differential expression of adaptive genes is under selection. Therefore, we validated eight potential reference genes from a panel of primer sets that were originally designed for *Mus musculus *and tested their application for relative gene expression analysis in *A. flavicollis*.

## Results and discussion

### Potential reference genes

All 15 tested reference gene primer sets were originally designed for *Mus musculus *(Table 1). It turned out that none of the six primer sets from the RTPrimer data base [17] nor the primers for the reference gene *B2 m *of the Mouse Normalisation Gene Panel (Quantace) did amplify a product in the related non-model species *Apodemus flavicollis*. Transferring primer sets from closely related organisms limits the set of genes that are tested and might reduce the chance to find a good internal reference as the possible choice depends on the set and number of genes that were used. However, eight intron spanning primer sets of the Mouse Normalisation Gene Panel (Quantace) performed well in *A. flavicollis*, which still is a comparable number to other validation studies [9,26-28]. They amplified conserved parts of the succinate dehydrogenase complex (*Sdha*), γ-actin (*Actg1*), ribosomal protein S18 (*Rps18*), ribosomal protein L13a (*Rpl13a*), phosphoglycerate kinase 1 (*Pgk1*), calnexin (*Canx*), β-actin (*Actb*) and ubiquitin C (*Ubc*). Further functions and accession numbers are provided in Table 1. As the sequences of the commercial primer sets were unknown we applied molecular cloning and subsequent sequence analysis using the vector primers T7 and M13 to confirm amplicon identity. The GenBank accession numbers are provided in Table 2. All gene identities could be confirmed but *Rpl13a *turned out to be not intron spanning. Sequencing revealed that the commercial primer set for *RPL13a *did amplify part of the small nuclear RNA (sno RNA) *U35 *that is situated in the sixth intron of *Rpl13a *and part of the seventh exon of *Rpl13a*.

**Table 1:** Names, function, database ID and annealing temperature (Ta) of the tested primer sets

Abbreviation	Gene	Function	Accession Number	T_a _[°C]
*Actb-1*	actin, beta	cytoskeletal structural protein	2848^#^	60°C
***Actb-2***	actin, beta	involved in cell motility, structure and integrity	ensmusg00000029580^+^	55°C
***Actg1***	actin, gamma, cytoplasmic1	Cytoskeletal structural protein	ensmusg00000062825^+^	55°C
B2 m-1	beta-2 microglobulin	cytoskeletal protein involved in cell locomotion	3584^#^	60°C
B2 m-2	beta-2 microglobulin	cytoskeletal protein involved in cell locomotion	ensmusg00000060802^+^	55°C
***Canx***	calnexin	protein folding and quality control in the endoplasmic reticulum	ensmusg00000020368^+^	55°C
Gapdh	glyceraldehyde-3-phosphate dehydrogenase	carbohydrate metabolism	3244^#^	60°C
Hprt1	hypoxanthine guanine phosphoribosyl transferase 1	metabolic salvage of purines in mammals	50^#^	55°C
***Pgk1***	phosphoglycerate kinase 1	transferase enzyme in the glycolysis	ensmusg00000062070^+^	55°C
***Sdha***	succinate dehydrogenase complex, subunit A	tricarboxylic acid cycle	ensmusg00000021577^+^	55°C
***Rpl13a***	ribosomal protein L13A	member of ribosome protein	enst00000270634^+^	55°C
Rplp0	ribosomal protein, large, P0	member of ribosome protein	2861^#^	60°C
***Rps18***	ribosomal protein S18	member of ribosome protein	ensmusg00000008668^+^	55°C
Tuba1a	tubulin, alpha 1A	structural protein	1484^#^	58°C
***Ubc***	Ubiquitin C	protein degradation	ensmusg00000008348^+^	55°C

The eight reference genes that performed well in *A. flavicollis *liver samples are marked in bold.

^# ^RTPrimerDB: http://medgen.ugent.be/rtprimerdb

^+ ^Ensembl Project: http://www.ensembl.org/index.html; Primer of Gene Normalization Panel

**Table 2:** Descriptive statistics of the tested reference genes

	*Rps18*	*Sdha*	*Canx*	*Pgk1*	*Actg1*	*Ubc*	*Rpl13a*	*Actb*
**GenBank ID**	GU188049	GU188053	GU188051	GU188052	GU188050	GU188054	GU188056	GU188055

**AM_amplification rate_**	1.86	1.88	1.83	1.85	1.88	1.86	1.85	1.82
**CV_amplification rate_**	0.06	0.05	0.05	0.05	0.06	0.05	0.05	0.05

**GM_Ct-value_**	14.62	15.37	16.63	15.72	16.83	15.72	26.05	16.05
**AM_Ct-value_**	14.64	15.42	16.68	15.78	16.88	15.79	26.10	16.16
**CV_Ct-value_**	0.04	0.07	0.07	0.07	0.07	0.08	0.06	0.09
**Minimum_Ct-value_**	13.86	13.90	14.65	13.42	14.94	12.87	24.30	13.64
**Maximum_Ct-value_**	16.74	17.68	19.61	18.62	19.68	18.26	28.87	20.50
**SD_Ct-value_**	**0.65**	**1.09**	**1.09**	**1.10**	1.11	1.26	1.45	1.49
**SD-threshold**	1.12	1.10	1.15	1.13	1.10	1.12	1.13	1.16

**Minimum [x-fold]**	-1.60	-2.52	-3.30	-4.10	-3.29	-5.90	-2.94	-4.24
**Maximum [x-fold]**	3.71	4.27	6.10	5.93	6.00	4.84	5.65	14.49
**SD [± x-fold]**	1.50	1.99	1.99	2.00	2.01	2.21	2.49	2.56

Arithmetic mean (AM), geometric mean (GM), coefficient of variance (CV) and standard deviation (SD) of the amplification rate E and the Ct-values for every potential reference gene. The genes are ordered by their *SD*_*Ct*-*value*_. Genes that showed a *SD*_*Ct*-*value *_smaller than the SD-threshold are considered to be suitable reference genes and marked in bold. The last three rows show the maximum and minimum values of the over- and under-expression of a gene in relation to its calculated geometric mean (displayed as x-fold ratio) as well as the standard deviation (calculated with BestKeeper).

### Amplification rate

The average arithmetic mean (AM) of the amplification rate *E *ranged from 1.82 for *Actb *to 1.88 for *Actg1 *(Table 2). The coefficient of variance (CV) expresses the variance of the amplification rate between the different qPCR runs. It was 0.05 for all reference genes except for *Actg1 *and *Rps18 *(0.06) (Table 2). The lowest Ct -value recorded was 12.87 cycles and the highest was 28.87 cycles. The difference in the Ct -values between the genes within a run ranged from 9.83 cycles to 14.81 cycles (Table 2).

### Identification of optimal reference genes

All our analyses on the stability of the references genes using the different algorithms showed consistent results with only slight differences in the ranking order (Table 3). A Kendall's *W *test showed a very high concordance of gained orders (Kendall's *W *= 0.958, *χ*^2 ^= 20.108, df = 7, p < 0.01). The resulting mean rank order of the genes from low to high variation was *Rps18*, *Sdha*, *Canx*, *Actg1*, *Pgk1*, *Ubc*, *Rpl13a *and *Actb*.

**Table 3:** Ranking order of the reference genes obtained by the three used algorithms implemented in BestKeeper, NormFinder and geNorm.

	*Rps18*	*Sdha*	*Canx*	*Pgk1*	*Actg1*	*Ubc*	*Rpl13a*	*Actb*
**BestKeeper**	1	2	3	4	5	6	7	8
**NormFinder**	2	1	3	4	5	7	6	8
**geNorm**	1.5	3	1.5	4	5	6	7	8

**mean rank**	1.5	2	2.5	4	5	6.3	6.7	8

### BestKeeper analysis

The software BestKeeper ranked all genes by their Ct-value variance (low to high): *Rps18*, *Sdha*, *Canx*, *Pgk1*, *Actg1*, *Ubc*, *Rpl13a *and *Actb *(Table 2). It considers all genes showing a variation in their amount of starting material by the factor two or more as unstable [14]. In an ideal PCR reaction with an amplification rate of two (100% reaction efficiency) this would be any gene whose Ct-values show a standard deviation *SD*_*Ct*-*value *_> 1, which is used as default by BestKeeper. Hibbeler *et al*. [8] already ruled out that the default setting of BestKeeper might be a too strict rule and limits its use to a very restricted experimental setup. In in-vivo samples, it is difficult to achieve a *SD*_*Ct*-*value *_< 1 as whole-tissue biopsies usually represent a composition of different cell types and show therefore a higher variation [29]. Additionally in biological samples the reaction efficiency is rarely 100% [13]. We therefore adjusted the SD-threshold for each gene to its specific efficiency. As a consequence we made BestKeeper more applicable but still rejected every gene whose *SD*_*Ct*-*value *_indicated a variation in the starting template by the factor two. According to our study the first four genes could be considered as stable reference genes as the *SD*_*Ct*-*value *_was lower than their individual SD-threshold whereas the other genes were considered as unstable (Table 2).

### NormFinder analysis

**Figure 1:**
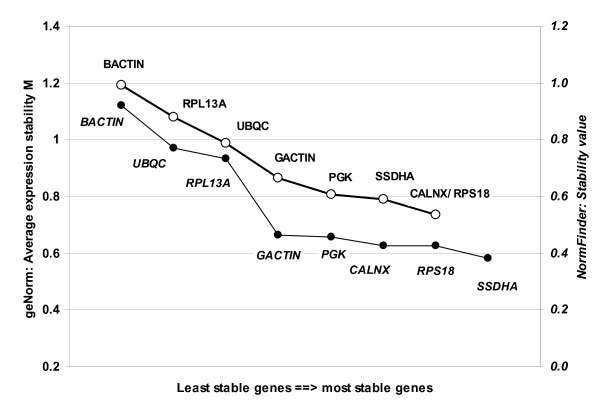
**Gene expression stability values of the eight potential reference genes**. The stability values on the right axis were calculated with NormFinder [12] (black circles) and the average expression stability values M (white circles) on the left axis were calculated with geNorm [6] after stepwise exclusion of the least stable gene. Genes are plotted from the least to the most stable expressed genes.

The ranking of the computer program NormFinder [12] is not based on the Ct-values but on the expression values. Compared to the BestKeeper ranking only two changes at the first and the sixth position occurred: *Sdha *(<0.382, Fig. 1) changed place with *Rps18 *<0.427) and was the most stable gene while *Rpl13a *(<0.734) changed place with *Ubc *(>0.771) and became the sixth most stable gene. However, the five most stable genes differ only by just 0.084 points in their stability values, while the difference among the last three genes is more than three times larger than this (Fig. 1).

### geNorm analysis

The program geNorm [6] ranks the potential reference genes due to their average pairwise variation in expression of one gene compared to each other gene of the set. It is independent of inter-run variability or different reverse transcription RT efficiencies. Only one change occurred compared to the ranking of BestKeeper: *Canx *becomes together with *Rps18 *one of the two most stable genes, which cannot be further ranked (M_Canx/Rps18 _= 0.73) (Fig. 1). Whereas geNorm is susceptible to identify co-regulated genes as optimal reference genes as they would show a constant ratio, NormFinder and BestKeeper do not suffer from this problem. As all three softwares produce consistent results we assume that the potential problem of co-regulated genes does not apply to our data.

### Number of reference genes

**Figure 2:**
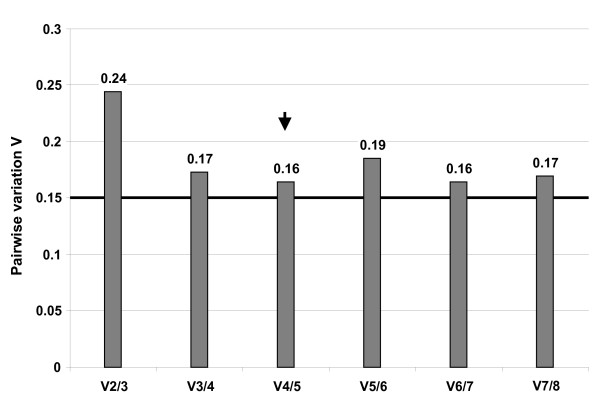
**Pairwise variation *V*_*n*/*n*+1 _between the normalizing factors *NF*_*n *_and *NF*_*n*+1_The variation *V*_*n*/*n*+1 _between *NF*_*n *_and *NF*_*n*+1 _was calculated with geNorm to determine the optimal number of reference genes that should be used for normalization**. The empirical cut-off value 0.15 defined by Vandensompele *et al*. [6] is marked by a thick line. The lowest variability is marked with an arrow.

The use of just a single reference gene may result in a more than 6-fold erroneous normalization [6] and it is therefore recommended to use more than one reference gene [1,30] and calculate a normalization factor (NF) [6,14]. As Vandesompele *et al*. [6] pointed out it is a trade off between accuracy and feasibility, but it seems inappropriate if the number of reference genes exceeds the number of genes of interest by far. To find the optimal number of reference genes for normalization geNorm calculates whether the stepwise inclusion of a less stable gene into the normalization factor *NF*_*n *_affects the variance *V*_*n*/*n*+1 _compared *NF*_*n*+1 _(Fig. 2). We observed the lowest Variation *V*_*n*/*n*+1 _between inclusion of the fourth and fifth most stable reference gene (*V*_4/5 _= 0.164) (Fig. 2). A high *V*_*n*/*n*+1 _means that the inclusion of the next gene had a big effect and it still should be included into the calculation of an accurate *NF*. *V*_4/5 _= 0.164 is a bit higher than the cut off value of 0.15 suggested by Vandesompele *et al*. [6]. But this is an empirical value and should not be taken as a too strict cut off value, as it is already suggested by the geNorm manual itself. Although *Actg1 *was refused as a reference gene by BestKeeper analysis we would suggest to use the first five reference genes *Rps18*, *Canx*, *Sdha*, *Pgk1 *and *Actg1 *for calculating a *NF *in *A. flavicollis*, as *Actg1 *only slightly missed the SD-threshold. This is further supported by the results of NormFinder as we observed a clear increase of the stability value between the fifth and the sixth most stable gene. This increase is more then three times as high as the over-all difference between the first and the fifth gene. This shows that the first five genes are much more similar in expression stability than the last three ones.

## Conclusions

Although we expected higher expression variability due to more heterogeneity in terms of age or physiological stages in our samples we could show that relative quantification via real-time PCR is feasible in samples from wild caught animals. The five genes *Rps18*, *Canx*, *Sdha*, *Pgk1 *and *Actg1 *were most stable and should allow an appropriate normalization factor for accurate measurement. We hope that our study will encourage other researchers to apply qPCR in eco-genomic studies on other wildlife species.

## Methods

### Sample collection

We live trapped wild yellow necked mice (*Apodemus flavicollis*) in 2007/08 in a deciduous forest about 35 km north-east of Hamburg, Germany. Mice were anesthetized by inhalation of isoflurane (Forene^©^) and then sacrificed immediately by cervical dislocation at the trapping site. Liver samples were taken and stored in RNA-Later (Sigma), kept at 4°C for 24 h and then frozen at -20°C until further treatment.

### RNA extraction and cDNA synthesis

Thirty mg liver tissue of 14 animals were placed in tubes with 500 μl of QIAzol lyses reagent (Qiagen) with 1.4 mm ceramic beads. Tissue was disrupted in a homogenizer (Precellys, Bertin Technologies) (2 × 10 s at 5000 rpm) and total RNA was extracted following the QIAzol lyses reagent protocol and dissolved in 87.5 μl of water. A DNA digestion with DNase I (RNase-free DNase Kit, Qiagen) and a subsequent clean-up via RNeasy spin columns (Qiagen) according to the manufacturer's protocol was done. Total RNA was finally eluted in 60 μl of water and its amount and purity was assessed with the Nanodrop 1000 (Thermo Scientific) three times and averaged. Two μg of total RNA were reverse transcribed with Oligo-dT_18 _primers (5 μM). Reverse transcription was run in triplicates of 40 μl using the SensiMix two step kit (Quantace) according to the manufacturer's protocol. All RT-triplicates were mixed and the copied cDNA was diluted **1**:16 prior qPCR with aqua dest.

### Primer selection

We chose six rodents primer sets out of the RTPrimer data base because they potentially amplified reference genes with similar length and identical annealing temperature *T*_*a*_. We also tested nine intron spanning primer sets out of the commercially available Mouse Normalisation Gene Panel (Quantace) (Table 1). All these potential reference gene primer sets were originally designed for the model organism *Mus musculus *and we applied them to our non-model organism *A. flavicollis*.

### Quantitative real-time RT PCR

SYBR green qPCR was performed with SensiMix two step kit (Quantace) in a 25 μl volume on a Rotor Gene 3000 (Corbett Research). All qPCR reactions were run in triplicates with a no-template control to check for contaminations. Each tube contained 4 μl of cDNA template, 12.5 μl SensiMix dT (Quantace), 0.5 μl SYBR Green solution, 0.5 μl primer (50 μM) and 7.5 μl dH_2_O. The qPCR conditions were 10 min at 95°C and 45 cycles of each 95° for 15 s, 55°C for 20 s and 72°C for 20 s. Melting curve analysis was performed from 65° to 95°C in 0.5°C steps each lasting 5 s to confirm presence of a single product and absence of primer-dimers. The individual amplification rate *E *for every single reaction tube was determined by the 'comparative quantification' function (Corbett Software 6.1.81) to avoid inter-run variation. *E *is defined as the average increase of fluorescence in the raw data for five cycles following the 'Takeoff' value. This Takeoff value is specified as the time at which the second derivative of the raw data is at 20% of its maximum (Corbett Software 6.1.81). This point marks the end of the background noise and indicates the transition into the exponential phase of the reaction. *E *was averaged for each gene out of the three replicates in each run. To normalize the raw data the individual background fluorescence from cycle one to the Takeoff value was averaged and all data points of a sample were divided by this average background level ('Dynamic Tube' function, Corbett Software 6.1.81). Individual threshold cycle values (Ct-values) were obtained by setting a threshold manually at 0.01 of the normalized fluorescence ignoring the first five cycles. The Ct-values for a gene were averaged for the three replicates in each run. We calculated the expression of each gene arbitrarily as *Q *= *E*^-*Ct *^. Note that *Q *is not the real amount of DNA copies *N*_*t *_= *N*_0 _*E^*t *^to a time point t but rather the fluorescence that is measured proportional to *N*_*t*_. As we set a certain fluorescence threshold we set . With the known *E *and the Ct-value the ratio between two genes depends only upon their start amount of cDNA *N*_0_.

### Determination of reference gene expression stability

The stability of the selected reference genes was determined by BestKeeper [21], NormFinder [18] and geNorm [12]. Concordance between their different ranking orders was tested with Kendall's *W *implemented in SPSS 16.0.2.

BestKeeper ranks the reference genes by the variation of their Ct-values. The gene with the lowest standard deviation (SD_Ct-value_) is proposed to be the most suitable reference gene. Like BestKeeper, we excluded every gene showing a SD_Ct-value _that would result in a variation of the starting material by the factor two. But unlike BestKeeper, we calculated this SD-threshold for each gene based on its known over-all run average *E*: .

NormFinder [18] instead uses a model based approach to analyse the variance in the expression data. It allows for intra- and intergroup variation which makes it more robust against co-expressed genes. In this experiment it was not necessary to distinguish between intra- and intergroup variation as we had only one group of samples. NormFinder calculates a stability value for each gene and the gene with the lowest value is supposed to be the most stable out of the tested set of genes.

GeNorm [12] bases on the simple assumption that expression of two ideal reference genes will always have the same ratio among samples regardless of the experimental conditions before the real-time PCR. The ratio between two genes (*Y *and *X*) in a sample is . The average expression stability value M for each gene is calculated using the expression data. M is the average pairwise variation of a gene compared with each of the other potential reference genes in one sample. The average M of all genes together is then calculated by stepwise exclusion of the least stable gene until the two most stable genes of the set remain that can not be ranked any further.

GeNorm also allows estimating the optimal number of reference genes which should be used for normalization. It calculates the normalization factor (*NF*) based on the geometric mean of the expression of more than one reference gene. The more reference genes included in this *NF *the less possible outliers account. On the other hand using to many genes might include unstable reference genes making it less accurate. GeNorm calculates the *NF*_*n *_for the two most stable reference genes based on the geometric mean of the expression data and then the *NF*_*n*+1 _with the next most stable gene. To determine how many genes should be used for accurate normalization the pairwise variation *V*_*n*/*n*+1 _was determined out of two sequential normalization factors (*NF*_*n *_and *NF*_*n*+1_).

All research reported in this manuscript adhered to the legal requirements of Germany were and complied with the protocols approved by the responsible state office for Agriculture, Environment and Rural Areas of Schleswig-Holstein (Referenz No: LANU 315/5327.74.1.6).

## Competing interests

The authors declare that they have no competing interests.

## Authors' contributions

JA performed the sample collection, data acquisition, analysis and interpretation as well as drafting the manuscript. SS was responsible for the overall study design, supervised the study and helped to draft the manuscript. Both authors read and approved the final manuscript.
